# Therapeutic efficacy of artesunate-amodiaquine and artemether-lumefantrine and polymorphism in *Plasmodium falciparum kelch13-propeller* gene in Equatorial Guinea

**DOI:** 10.1186/s12936-021-03807-x

**Published:** 2021-06-22

**Authors:** Matilde Riloha Rivas, Marian Warsame, Ramona Mbá Andeme, Salomón Nsue Esidang, Policarpo Ricardo Ncogo, Wonder Philip Phiri, Consuelo Oki Eburi, Corona Eyang Edú Maye, Didier Menard, Eric Legrand, Pedro Berzosa, Luz Garcia, Angela Katherine Lao Seoane, Spes Caritas Ntabangana, Pascal Ringwald

**Affiliations:** 1 National Malaria Control Programme, Ministry of Health and Social Welfare, Malabo, Equatorial Guinea; 2 School of Public Health and Community Medicine, University of Gothenburg, Gothenburg, Sweden; 3 State Foundation, Health, Children and Social Welfare (FCSAI), Madrid, Spain; 4 Medical Care Development International, Malabo, Equatorial Guinea; 5 Malaria Genetics and Resistance Unit, INSERM U1201, Institut Pasteur, Paris, France; 6 Malaria and NTDs Laboratory, National Centre of Tropical Medicine, Institute of Health Carlos III, Madrid, Spain; 7 World Health Organization, County Office Equatorial Guinea, Malabo, Equatorial Guinea; 8 World Health Organization, IST for Central Africa, Libreville, Gabon; 9 World Health Organization, Headquarters, Geneva, Switzerland

**Keywords:** Artesunate-amodiaquine, Artemether–lumefantrine, *Plasmodium falciparum*, Efficacy, Equatorial Guinea

## Abstract

**Background:**

Artesunate-amodiaquine (ASAQ) and artemether-lumefantrine (AL) are the currently recommended first- and second-line therapies for uncomplicated *Plasmodium falciparum* infections in Equatorial Guinea. This study was designed to evaluate the efficacy of these artemisinin-based combinations and detect mutations in *P. falciparum kelch13*-propeller domain gene (*Pfkelch13*).

**Methods:**

A single-arm prospective study evaluating the efficacy of ASAQ and AL at three sites: Malabo, Bata and Ebebiyin was conducted between August 2017 and July 2018. Febrile children aged six months to 10 years with confirmed uncomplicated *P. falciparum* infection and other inclusion criteria were sequentially enrolled first in ASAQ and then in AL at each site, and followed up for 28 days. Clinical and parasitological parameters were assessed. The primary endpoint was PCR-adjusted adequate clinical and parasitological response (ACPR). Samples on day-0 were analysed for mutations in *Pfkelch13* gene.

**Results:**

A total 264 and 226 patients were enrolled in the ASAQ and AL treatment groups, respectively. Based on per-protocol analysis, PCR-adjusted cure rates of 98.6% to 100% and 92.4% to 100% were observed in patients treated with ASAQ and AL, respectively. All study children in both treatment groups were free of parasitaemia by day-3. Of the 476 samples with interpretable results, only three samples carried non-synonymous *Pfkelch13* mutations (E433D and A578S), and none of them is the known markers associated with artemisinin resistance.

**Conclusion:**

The study confirmed high efficacy of ASAQ and AL for the treatment of uncomplicated falciparum infections as well as the absence of delayed parasite clearance and *Pfkelch13* mutations associated with artemisinin resistance. Continued monitoring of the efficacy of these artemisinin-based combinations, at least every two years, along with molecular markers associated with artemisinin and partner drug resistance is imperative to inform national malaria treatment policy and detect resistant parasites early.

*Trial registration* ACTRN12617000456358, Registered 28 March 2017; http://www.anzctr.org.au/trial/MyTrial.aspx

## Background

Countries in the World Health Organization (WHO) African Region bear most of the malaria burden and account for 94% of the estimated global malaria cases (213 million cases) and deaths (380,000) in 2019 [1]. Providing effective treatment to patients suffering from uncomplicated falciparum malaria prevents progression of the disease to a severe form or death and consequently reduces mortality and disease burden. *Plasmodium falciparum* resistance to anti-malarial drugs poses a constant threat to the successful treatment of malaria infections. The emergence and spread of chloroquine and sulfadoxine/pyrimethamine resistances have led the WHO to recommend artemisinin-based combination therapy (ACT) for the treatment of uncomplicated falciparum infection [2]. ACT combines potent and fast-acting artemisinin derivatives with a long and slow-acting partner drug able to clear residual parasitemias. The currently recommended artemisinin-based combinations are artemether-lumefantrine (AL), artesunate-amodiaquine (ASAQ), artesunate-sulfadoxine/pyrimethamine (ASSP), artesunate-mefloquine (ASMQ), and dihydroartemisinin-piperaquine (DHAPPQ) and artesunate-pyronaridine (ASPY) [3].

Improved access to effective antimalarial treatments has contributed significantly to a marked reduction in the burden of malaria in recent years [1]. AL followed by ASAQ are the most commonly recommended first and/or second line anti-malarial drugs in malaria endemic countries in Africa [1] and they remain efficacious after more than a decade of use [3]. However, the efficacy of ACT is threatened by the recent emergence of parasites resistant to artemisinin and partner drugs. Point mutations in the *P. falciparum kelch 13* (*Pfkelch13*) propeller domain gene have been found to confer artemisinin partial resistance expressed by delayed parasite clearance [4, 5]. Since 2012, resistance to both artemisinin and partner drugs has been observed in several countries in Southeast Asia [5–9] leading to treatment failure with artemisinin-based combinations, such as DHA-PPQ [10–13]. Moreover, and more worryingly, mutants *Pfkelch13* parasites (R561H) confering artemisinin partial resistance has been shown to emerge in 2014–2015 in one site in Rwanda followed by its expansion in another site located apart 100 km, several months later [14]. These findings pose a real threat to malaria case management, a fundamental component of current malaria intervention, and make regular monitoring of the efficacy of ACT, as recommended by the WHO [3], essential to support timely review of malaria treatment guidelines and ensure that malaria patients receive efficacious treatment [3].

In Equatorial Guinea, malaria is a major health problem for the entire population (1,355,982) with an estimated 321,438 (186,000–524,000) cases and 652 (440–1000) deaths in 2019 [1]. The majority of the disease burden occurs on the mainland, where children bear the brunt of the disease [15]. Equatorial Guinea recommended ASAQ as first-line drug in 2008 and AL as second-line drug in 2010 for the treatment of uncomplicated falciparum infection [16]. Since the introduction of these artemisinin-based combinations in the country, only one study (unpublished) has been conducted to evalute the efficacy of ASAQ, which showed PCR corrected cure rate > 96.6% [17]. Recently, *PfK13* nonsynonimous mutation (M579I), associated with delayed parasite clearance and increased in vitro parasite survival rate as measured by the Ring-stage Survival Assay [18] has been detected in a Chinese traveller returning from Equatorial Guinea [19]. The current study evaluated the efficacy of ASAQ and AL as well as the detection of mutations in the *Pfkelch13* gene to inform national malaria treatment policy.

## Methods

### Study area, design and population

**Fig. 1:**
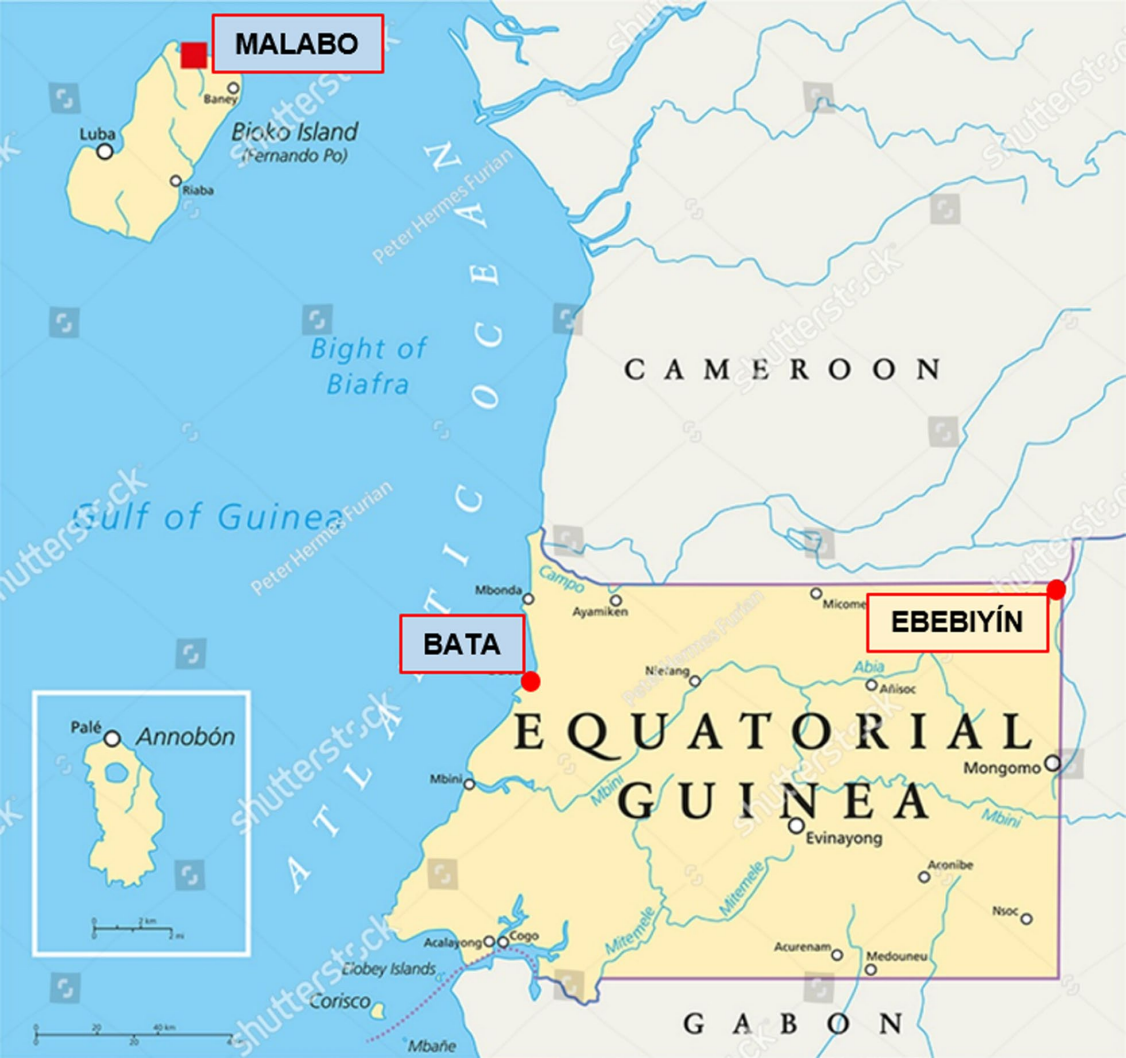
Map of Equatorial Guinea showing the study sites

Equatorial Guinea is located in Central Africa and is divided into two regions: the mainland area, which lies between Cameroon and Gabon, and the island region (Bioko, Annobo'n and Corisco Bay). About 75% of the country's population lives in the mainland. The study was conducted in selected health facilities in three study sites: Malabo in Bioko Island and Bata and Ebebiyin in Litoral and Kié-Ntem provinces in the mainland, respectively (Fig. 1). The health facilities in the study were Regional Hospital in Malabo, Provincial Hospital and Angokong Health Centre in Ebebiyin, Regional Hospital, Maria Rafols Health Centre and Maria Gay Health Centre in Bata. The study was a single arm cohort study that investigated the clinical therapeutic efficacy of ASAQ and AL in the treatment of uncomplicated falciparum infection. Children with uncomplicated falciparum malaria who met the study inclusion criteria were enrolled sequentially, first to ASAQ until sample size was reached and then to AL in each site, and assessed clinically and parasitologically for 28 days, based on the 2009 WHO protocol [20].

### Recruitment procedure and treatment

**Fig. 2:**
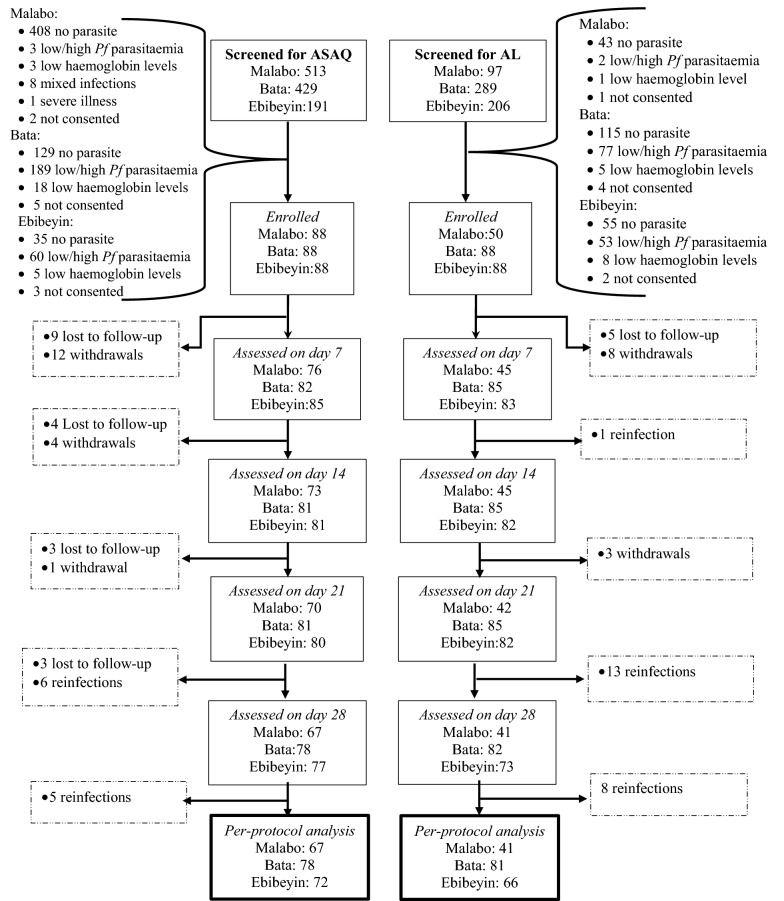
Study profile: enrolment and follow-up. *ASAQ* artesunate-amodiaquine, *AL* artemether-lumefantrine, *Pf* *Plasmodium falciparum*

Potential study children attending study health facilities from August 2017 to July 2018 were screened and enrolled if they met the following eligibility criteria: age between 6 months and 10 years, axillary temperature ≥ 37.5 °C and/or history of fever in the past 24 h, *P. falciparum* monoinfection, parasitaemia of 500 to 200,000 asexual parasites/µl, willingness to comply with the study visit schedule, and informed consent from parents or guardians (Fig. 2). Children with exclusion criteria, including the presence of general danger signs or evidence of severe falciparum malaria, mixed or monoinfection with non-falciparum species, severe malnutrition, febrile conditions due to diseases other than malaria (measles, acute lower respiratory tract infection, severe diarrhea with dehydration), or known underlying chronic diseases (e.g., cardiac, renal, or liver disease, HIV/AIDS), received appropriate care and treatment according to national guidelines. In addition, children on regular medication that could have affected the pharmacokinetics of the study ACT and those with a history of hypersensitivity reactions to the medications were excluded.

Children in the ASAQ group received daily dose of ASAQ for three consecutive days based on recommended weight ranges: one tablet and two tablets of 25 mg artesunate/67.5 mg for children weighing ≥ 4.5 to < 9 kg and 9 to < 18 kg, respectively; one tablet and two tablets of 100 mg artesunate/270 mg amodiaquine for children weighing 18 to < 36 kg and ≥ 36 kg, respectively. For children treated et al. doses of AL were administered twice daily for 3 days according to recommended weight: one tablet for children weighing 5–14 kg, two tablets for 15–24 kg, and three tablets for 25–34 kg. All treatment doses were administered under direct observation by the study team and patients were observed for 30 min. If the first dose was vomited, the treatment dose was administered again. If vomited again, the patient received an artesunate injection according to national guidelines and the patient was withdrawn from the study. Study drugs were obtained from the WHO.

Enrolled children were followed for up to 28 days at scheduled visits on days 1, 2, 3, 7, 14, 21, and 28, and at unscheduled visits if symptoms worsened or recurred. Clinical and parasitological examinations were performed at each visit. If the parents/caregivers did not show up for the scheduled visit, a team member visited them at home.

### Sample size estimation

A minimum sample of 73 children per drug per site was estimated, based on the assumption of a 5% treatment failure rate for each drug ASAQ and AL with a 95% confidence level and 5% precision. An additional 20% was added to allow for "lost to follow-up" and withdrawal during the 28-day follow-up period. The target sample size was 88 patients per drug per site.

### Malaria microscopy

Thick and thin blood slides were stained with Giemsa and examined microscopically to detect malaria parasites and determine level of parasitaemia based on the WHO procedure [20]. The number of asexual parasites was counted against 200 white blood cells (or per 500 if the count was < 100 parasites/200 white blood cells). A blood slide was confirmed negative if no parasite was seen after counting 1000 white blood cells. Parasite density, defined as parasites per µl, was calculated assuming a leukocyte count of 6000/µl of blood. All blood slides were examined by two independent microscopists. A third microscopist re-examined the slides with discordant results in terms of species diagnosis, parasite density of > 50% or presence of parasites. Final parasite density was calculated by averaging the two closest counts.

### Genotyping of malaria parasite

Filter paper blood samples were collected from each patient on day-0 and in case of recurrence, on the day of parasite recurrence (from day-7). Samples were dried and stored in individual plastic bags containing desiccant. Each dried blood spot was punched out with a sterile puncher, and the spots were placed in numerical order in a 96-well plate. Parasite DNA was extracted using QIAamp DNA Blood Mini Kit (Qiagen). The DNA samples (day-0 and day of recurrence) were analysed for genotyping of the highly polymorphic regions *msp1, msp2* (*merozoite surface proteins 1* and *2*) and *glurp* (*glutamate-rich protein*) loci, as described elsewhere [21]. The genotypic profiles of the parasites at day-0 and day of recurrence were compared to determine whether the recurent infections were a recrudescence (same strain) or a new infection (different strain), according to the current WHO-recommended algorithm [22]. As an explanatory endpoint, reinfection and recrudescence were also determined by the newly proposed two out of three (2/3) algorithm [23]. In this strategy, the classification of recurrent failures is based on a consensus result of *msp1* and *msp2* and disparate results are resolved by *glurp*. Such an analysis demands concommittant results from at least two markers for classification of reinfection or recrudescence compared to three with the standard WHO methodology. If results for only 2 markers were available and results for a third marker were missing, the PCR correction was classified as undertermined. Samples were analysed at Institut Pasteur in Paris, France.

### Molecular markers of artemisinin resistance

DNA extracted from day-0 dried blood spots were analysed to detect the presence of mutations in the propeller domain of *Pfkelch13* gene (PF3D7_1343700) previously described to be associated with artemisinin resistance [4]. PCR amplification (codons 440–680, 720 bp) was performed using the method described by Ariey et al*.* [4]. For the inner round, five μl DNA was amplified with 0.25 μM each primer, 0.2 mM dNTP, 2.5 mM MgCl2, and 1.25 U Taq DNA polymerase (Solis Biodyne, Estonia), in 25 μl volume using the following cycling program: 15 min at 95 °C, then 35 cycles of 30 s at 95 °C, 2 min at 58 °C, 2 min at 72 °C, and final extension 10 min at 72 °C. For the outer PCR round, 5 μl of primary PCR products were amplified under the same conditions, except for annealing and extension (1 min). PCR products were detected using capillary gel electrophoresis (Fragment Analyzer, Agilent, France). Double strand sequencing of PCR products was performed by Eurofins (Germany). Sequence polymorphisms were identified with the CLC Main Workbench 20 software (Qiagen) by using the 3D7 strain of *P. falciparum* (PF3D7_1343700) as a reference sequence. Electropherograms with mixed alleles were considered as mutant for the purpose of mutation frequency estimation. The quality control was assessed by including three blinded quality-control samples (wild type, C580Y and R539T) in each 96-well sequencing plate.

### Treatment response measure

Treatment response was classified as early treatment failure (ETF), late clinical failure (LCF), late parasitological failure (LPF) and adequate clinical and parasitological response (ACPR) before and after PCR correction using the WHO protocol [18]. The primary study endpoint was PCR-corrected adequate clinical and parasitological response. Secondary endpoints included parasitaemia at day-3 and PCR-uncorrected treatment failure.

### Ethical considerations

The study was approved by the Ministry of Health and Social Welfare of Equatorial Guinea (No. 731–150), the Ethics Committee of Spanish National Health Institute, Carlos III (CEI PI 57_2016-v3) and the WHO Research Ethics Review Committee (ERC.000286). Parents or guardians were informed about the study procedure, its benefits and potential risks, and their consent to enroll their children was obtained in writing before enrollment. They gave their written consent before enrolling their children in the study. If a patient, parent, or guardian was illiterate, he or she chose a witness to co-sign the consent form.

### Data analysis

Data were double entered, cleaned, and analysed using the software programme WHO excel (http://www.who.int/malaria/publications/atoz/9789241597531/en/). Enrolled patients who were lost to follow-up or withdrawn from the study, had recurrent parasitaemia with new infection, or unknown PCR (indeterminate or missing) were excluded from per-protocol analysis. However, these cases were included in the Kaplan–Meier analysis up to the day of loss or withdrawal from the study. Descriptive statistics including percentages, mean, standard deviation, and range were used. Student's t-test was used for analysis of continuous variables (parasite density and age) and Fisher's exact test was used for categorical data. A p-value of < 0.05 was considered significant.

## Results

### Baseline characteristics of enrolled children

Of the 1725 children screened, 490 were enrolled in the study: 264 were treated with ASAQ (88 in Malabo, 88 in Bata and 88 in Ebibeyin) and 226 with AL (50 in Malabo, 88 in Bata and 88 in Ebibeyin). The target sample size per drug and per site (n  =  88) was achieved except for the group treated with AL in Malabo site due to low malaria transmission. Baseline characteristics (age, axillary temperature, parasitaemia) of the study children in the different sites and treatment groups were comparable (Table 1).

**Table 1.** Baseline characteristics of the study children treated with artesunate-amodiaquine (ASAQ) or artemether-lumefantrine (AL)

Characteristic	Artesunate-amodiaquine			Artemether-lumefantrine		
	Malabo (N = 88)	Bata (N = 88)	Ebibeyin (N = 88)	Malabo (N = 50)	Bata (N = 88)	Ebibeyin (N = 88)
Males, n (%)	48 (54.5)	43 (48.9)	57 (64.8)	25 (50)	49 (55.7)	44 (50)
Mean age years (SD)^†^	4.8 (2.6)	4.2 (2.8)	2.6 (1.9)*	4 (2.1)	4.5 (2.9)	4.1 (2.4)
Axillary temperature (°C)						
Mean (SD)	37.5 (1.3)	37.3 (1)	38.1 (1.0)	38.1 (1)	38.0 (1.2)	38.3 (1.2)
Parasitaemia (per µl)						
Geometric mean	29357	38373	29240	37411	45528	40691
Range	500–200000	1770–200000	821–200000	1850–200000	3572–190500	4493–200000

### Treatment responses

Table 2 shows the treatment outcomes as per-protocol and Kaplan Meier analysis before PCR correction. For patients treated on ASAQ, per-protocol analysis of uncorrected PCR data showed ACPR of 100% (95% CI 94.6–100%), 96.3% (95% CI 89.6–99.2%) and 88.8% (95% CI 79.7–94.7%) in Malabo, Bata and Ebebiyin sites, respectively. Among patients treated with AL, per-protocol analysis of uncorrected PCR data revealed ACPR of 92.9% (95% CI 80.5–98.5%), 95.3% (95% CI 88.4–98.7%) and 73.5% (95% CI 62.7–82.6%) in Malabo, Bata and Ebebiyin, respectively. For the 41 paired samples, *msp1*, *msp2* and *glurp* results were available for 95.1%, 93.9% and 95.1%, respectively. Per-protocol analysis of PCR corrected data (using the WHO protocol) showed ACPR of 100% (95% CI 94.6–100%), 100% (95% CI 95.4–100%) and 98.6% (95% CI 92.5–100%) in patients treated with ASAQ in Malabo, Bata and Ebebiyin, respectively (Table 3). In children treated with AL, PCR corrected ACPR of 95.1% (95% CI 83.5–99.4%), 100% (95% CI 95.5–100%) and 92.4% (95% CI 83.2–97.5%) were in Malabo, Bata and Ebebiyin, respectively. Based on Kaplan Meier survival analysis, PCR-corrected cumulative cure rates varied from 98.7% to 100% for ASAQ and from 93.7% to 100% for AL (Table 3). Study children at the Ebebiyin site had a higher rate of new infections (9.1% and 19.3% in the ASAQ and AL groups, respectively), compared to less than 5% at the other sites (Table 3). However, the difference was significant only for the Ebebiyin group AL (Fisher's exact test: p  =  0.01). Children in both treatment groups were parasite free on day-2 except one case in the ASAQ group in Bata (1.2%), and all of them cleared parasitaemia by day-3.

**Table 2.** PCR-unadjusted treatment response of study patients treated with artesunate-amodiaquine (ASAQ) or artemether-lumefantrine (AL)

*PCR-unadjusted* Treatment responses	Artesunate-amodiaquine			Artemether-lumefantrine		
	Malabo (N = 88) n (%)	Bata (N = 88) n (%)	Ebibeyin (N = 88) n (%)	Malabo (N = 50) n (%)	Bata (N = 88) n (%)	Ebibeyin (N = 88) n (%)
LCF	0	1 (1.2)	2 (2.5)	0	0	0
LPF	0	2 (2.5)	7 (8.8)	3 (7.1)	4 (4.7)	22 (26.5)
ACPR	67 (100)	78 (96.3)	71 (88.8)	39 (92.9)	81 (95.3)	61 (73.5)
Total per-protocol	67	81	80	42	85	83
Lost follow-up/withdrawn	21 (23.9)	7 (8)	8 (9.1)	8 (16)	3 (3.4)	5 (5.7)
Kaplan Meier: cure rate	67 (100)	78 (96.3)	71 (88.8)	39 (92.9)	81 (95.3)	61 (73.5)

LCF: late clinical failure; LPF: late parasitological failure; ACPR: adequate clinical and parasitological response

**Table 3.** PCR-adjusted treatment response of study patients treated with artesunate-amodiaquine (ASAQ) or artemether-lumefantrine (AL)

Treatment responses	Artesunate-amodiaquine			Artemether-lumefantrine		
	Malabo (N = 88) n (%)	Bata (N = 88) n (%)	Ebibeyin (N = 88) n (%)	Malabo (N = 50) n (%)	Bata (N = 88) n (%)	Ebibeyin (N = 88) n (%)
*PCR-adjusted *WHO methodology						
LCF	0	0	0	0	0	0
LPF	0	0	1 (1.4)	2 (4.9)	0	5 (7.6)
ACPR	67 (100)	78 (100)	71 (98.6)	39 (95.1)	81 (100)	61 (92.4)
Total per-protocol	67	78	72	41	81	66
Lost follow-up/withdrawn	21 (23.9)	7 (8)	8 (9.1)	8 (16)	3 (3.4)	5 (5.7)
New infection	0	3 (3.4)	8 (9.1)	1 (2)	4 (4.5)	17 (19.3)
Kaplan Meier: cure rate	67 (100)	78 (100)	71 (98.7)	39 (95.2)	81 (100)	61 (93.7)
*PCR-adjusted *2/3 algorithm						
LCF	0	0	1 (1.3)	0	0	12 (16.4)
LPF	0	0	4 (5.3)	3 (4.9)	0	5 (7.6)
ACPR	67 (100)	78 (100)	71 (93.4)	39 (92.9)	81 (100)	61 (83.6)
Total per-protocol	67	78	76	42	81	73
Lost follow-up/withdrawn	21 (23.9)	7 (8)	8 (9.1)	8 (16)	3 (3.4)	5 (5.7)
New infection	0	2 (2.3)	2 (2.6)	0	2 (2.3)	7 (7.9)
Undetermined	0	1 (1.1)	2 (2.6)	0	2 (2.3)	3 (3.4)
Kaplan Meier: cure rate	100	100	93.6	92.9	100	84.5

Using the 2/3 algorythm, PCR correction remained the same in 22/41 (53.7%) recurrences, changed to recrudescence in 12/41 (29.2%) recurrences, and was undertermined for 7/41 (17.1%) recurrences (Table 3). The failure rate increased significantly in Ebibeyin for both treatment arms (5.2% for ASAQ and 8.8% for AL). No significant changes were observed for the other two sites.

### Artemisinin partial resistance marker

Among the 490 patients, three samples were missing and 11 gave non-interpretable results. Most of the samples with interpretable results (98.1%), carried *Pfkelch13* wild type allele and only three (0.6%) had non-synonymous mutations (two carried A578S and one with E433D, Table 4). None were previously linked with artemisinin resistance. Of the 8 patients with recrudescent parasite, only four samples were successfully sequenced, and all carried *Pfkelch13* wild type allele.

**Table 4.** Proportion of *Pfkelch13* alleles on pre-treatment samples with interpretable result

*Pfkelch13* allele	Malabo			Bata			Ebebiyin		Total	
	N		%	N		%	N	%	N	%
Wild type	131		100%	170		97.1%	168	98.8%	469	98.5%
Synonymous mutants										
R471R	0	0		2	1.2%		1	0.6%	3	0.6%
T478T	0	0		1	0.6%		0		1	0.2%
Non-synonymous mutants										
E433D	0	0		1	0.6%		0		1	0.2%
A578S	0	0		1	0.6%		1	0.6%	2	0.4%
Total	131	0		175	100%		170	100%	476	100%

## Discussion

Previous studies evaluating the efficacy of ASAQ in Equatorial Guinea reported a high cure rate with 97.3% ACPR in 2006 before the recommendation of ACT [24] and 96.6% ACPR four years later in 2010 [17]. The findings of the current study showed that patients treated with ASAQ achieved a high cure rate (PCR-adjusted ACPR), ranging from 98.6 to 100% across sites, demonstrating that it has maintained its efficacy after more than a decade of use in the country. The current study assessed the efficacy of AL for the first time in the country and showed similar high cure rate with a PCR-adjusted ACPR between 92 and 100%. Although, the cure rate of AL (92.4%) in Ebebiyin was above the threshold (< 90%) requiring for changing treatment policy [3], this data calls for close monitoring of the efficacy of this artemisinin-based combination. Overall, the findings are in line with reports from recent studies confirming that ASAQ and AL, the most commonly recommended ACTs for the treatment of uncomplicated falciparum infection, remain effective in Africa and support their continued use [25–44].

It is worth noting that malaria transmission varies in the study sites, with a low level in Malabo (Bioko Island) and a moderate to high level in the mainland where Bata and Ebebiyin sites are located [15]. A survey conducted in Bata district reported higher malaria prevalence (58.9%) in the rural setting compared to 33.9% in the urban area, where the study patients were recruited, [45]. Health facility based rapid assessment of malaria indicators in 2016 revealed a test positivity rate of 70.9% (2071/2872) and 34.9% (1635/4687) among consultations with suspected malaria at Angokong Health Centre and Provincial Hospital, respectively, in Ebiyein district (Riloha Rivas, pers. commun.). The ongoing high malaria transmission observed in Ebibeyin compared to the other sites may explain the higher rate of new infections detected using the standard WHO PCR correction method [22] and the higher treatment failure rate using the 2/3 algorithm [23], especially for AL, for which the partner medicines has a shorter half-life. In the absence of a gold standard tool to distinguish between reinfection and recrudescence, it is difficult to interpret this increase in treatment failure rate using the 2/3 algorithm. Nevertheless, it is surprising that two medicines with opposite mechanism of resistance could fail at the same site and at the same time [46].

All study patients cleared their parasitaemia by day 3, indicating the absence of delayed parasite clearance, and together with lack of the known *Pfkelch13* mutations associated with artemisinin resistance in South East Asia, may indicate the absence of partial artemisinin resistance in Equatorial Guinea. Ih addition, the data based on 476 clinical samples of *P. falciparum,* support that the indigenous M579I mutant, claimed to be associated with delayed parasite clearance and increased in vitro parasite survival rate [19], did not expand and spread across the country.

## Conclusion

The study confirmed that ASAQ and AL remain highly effective in treating uncomplicated falciparum infections more than a decade after their use in Equatorial Guinea and that there are no known *Pfkelch13* mutant parasites associated with artemisinin resistance. Continued monitoring of the efficacy of these artemisinin-based combinations, at least every 2 years, is imperative to inform national malaria treatment policy. In addition, recent evidence of the de novo emergence of the *Pfkelch13* mutation (R561H) associated with artemisinin resistance in Africa calls for monitoring molecular markers associated with artemisinin and partner drug resistance to detect resistant parasites early. In this study, the 2/3 algorithm increased the failure rate at high transmission site compared to the standard WHO methodology. Further comparison and validation in different transmission setting are needed before this new suggested algorithm can be systematically implemented for PCR correction.

## Data Availability

The dataset used in this study is available and can be shared upon reasonable request to NMCP through the corresponding author.

## Abbreviations

ACPR: Adequate clinical and parasitological response
ACTs: Artemisinin-based combination therapies
AL: Artemether–lumefantrine
ASAQ: Artesunate-amodiaquine
ASMQ: Artesunate-mefloquine
ASPY: Artesunate-pyronaridine
ASSP: Artesunate-sulfadoxine/pyrimethamine
DHAPPQ: Dihydroartemisinin-piperaquine
ETF: Early treatment failure
LCF: Late clinical failure
LPF: Late parasitological failure
msp1: Merozoite surface proteins 1
msp2: Merozoite surface proteins 2
glurp: Glutamate-rich protein
NMCP: National Malaria Control Programme
PCR: Polymerase chain reaction
WHO: World Health Organization
